# Chondroitin Sulfate Proteoglycan 4 and Its Potential As an Antibody Immunotherapy Target across Different Tumor Types

**DOI:** 10.3389/fimmu.2017.01911

**Published:** 2018-01-10

**Authors:** Kristina M. Ilieva, Anthony Cheung, Silvia Mele, Giulia Chiaruttini, Silvia Crescioli, Merope Griffin, Mano Nakamura, James F. Spicer, Sophia Tsoka, Katie E. Lacy, Andrew N. J. Tutt, Sophia N. Karagiannis

**Affiliations:** ^1^St. John’s Institute of Dermatology, School of Basic & Medical Biosciences, King’s College London & NIHR Biomedical Research Centre at Guy’s and St. Thomas’ Hospitals and King’s College London, Guy’s Hospital, London, United Kingdom; ^2^Breast Cancer Now Research Unit, School of Cancer and Pharmaceutical Sciences, King’s College London, Guy’s Cancer Centre, London, United Kingdom; ^3^Department of Informatics, Faculty of Natural and Mathematical Sciences, King’s College London, London, United Kingdom; ^4^School of Cancer and Pharmaceutical Sciences, King’s College London, Guy’s Cancer Centre, London, United Kingdom; ^5^Breast Cancer Now Toby Robins Research Centre, Institute of Cancer Research, London, United Kingdom

**Keywords:** CSPG4, MCSP, HMW-MAA, NG2, melanoma, triple-negative breast cancer, immunotherapy, antibodies

## Abstract

Overexpression of the chondroitin sulfate proteoglycan 4 (CSPG4) has been associated with the pathology of multiple types of such as melanoma, breast cancer, squamous cell carcinoma, mesothelioma, neuroblastoma, adult and pediatric sarcomas, and some hematological cancers. CSPG4 has been reported to exhibit a role in the growth and survival as well as in the spreading and metastasis of tumor cells. CSPG4 is overexpressed in several malignant diseases, while it is thought to have restricted and low expression in normal tissues. Thus, CSPG4 has become the target of numerous anticancer treatment approaches, including monoclonal antibody-based therapies. This study reviews key potential anti-CSPG4 antibody and immune-based therapies and examines their direct antiproliferative/metastatic and immune activating mechanisms of action.

## Introduction

Recent advances in the field of cancer immunotherapy, involving specific targeting modalities like monoclonal antibodies or chimeric antigen receptor (CAR) T cell therapies, depend on the identification of appropriate surface antigens. The search for appropriate targets is especially important for certain malignancies such as triple-negative breast cancer (TNBC), which lack expression of the human epidermal growth factor receptor 2 (HER2/*neu*), estrogen or progesterone receptors, thus rendering them insensitive to available targeted therapies.

Chondroitin sulfate proteoglycan 4 (CSPG4) is a highly glycosylated transmembrane protein, and a member of the chondroitin sulfate group of glycosaminoglycans (GAGs). CSPG4, also referred to as melanoma-associated chondroitin sulfate proteoglycan (MCSP), high-molecular-weight melanoma-associated antigen (HMW-MAA), or neuron-glial antigen 2 (NG2), was first associated with malignant melanoma and subsequently implicated in the pathology of other solid tumors of different origins, as well as of hematological cancers (1). It has been investigated as a potential immunotherapy target due to its restricted/low distribution in normal tissues and overexpression in certain tumors at different disease stages, and based on evidence for multiple roles in supporting tumor growth and dissemination. Together, long-emerging studies point to CSPG4 as a promising target for cancer therapies, including immunotherapies with monoclonal antibodies. In this review, we summarize reported functions of CSPG4 in cancer and we examine the development of ongoing immunotherapy strategies, most notably monoclonal antibodies that target CSPG4.

## CSPG4 Normal Tissue Distribution, Structure, and Physiological Functions

Chondroitin sulfate proteoglycan 4 is heterogeneously expressed on normal tissues such as mesenchymal stromal cells—adult progenitor cells, which have been suggested to lose its expression during terminal differentiation (2). Early immunofluorescence/immunohistochemisry data on the distribution of CSPG4 in normal tissues suggest it is expressed in nevi, epidermis and hair follicles but not detected in brain, thyroid, thymus, lung, liver, ureter, testis, spleen, ovary, or peripheral nerves (3). In another early study, immunohistochemical analysis of fetal and adult human tissues suggest CSPG4 distribution in the adrenal cortex, liver, choroid and small intestine in the fetus and in adult peripheral nerves, liver, salivary glands, bladder, lung bronchial glands and sebaceous glands (4). In more recent studies, expression of CSPG4 and its rat ortholog has been reported at low or moderate levels on neuronal glial cells, arteriolar pericytes, smooth muscle cells, macrophages, melanocytes, articular chondrocytes, and others (5–11). At the RNA level, CSPG4 expression has been recently reported in skin, trachea, veins, lung, heart, muscle, diaphragm, adipose tissue, uterus, prostate, thymus, spleen, bone marrow, and gastrointestinal tissue, but importantly at 6.6 times lower levels than in tumors (12).

Its physiological functions are not completely understood and multiple studies report specific roles in different tissues throughout development. In placenta formation, the expression of CSPG4 on extravillous trophoblasts has been implicated in their differentiation and migration (13). CSPG4 is also proclaimed to be involved in angiogenesis and vascularization. Using murine *in vivo* models, NG2 was shown to induce *de novo* vascularization of otherwise the avascular corneal tissue, suggesting an important role in angiogenesis (14). Further reports suggest the involvement of CSPG4 in glial and oligodendrocyte formation and neuronal network regulation, epithelial keratinocyte replenishment, and epidermal stem cell positioning and homeostasis (15, 16).

Although a full understanding of the physiological roles of CSPG4 is still required, all reports suggest it is ubiquitously involved in multiple tissue development and homeostasis processes, and its roles may be differentially modulated based on the nature of the local tissue microenvironment (17). The regulation of CSPG4 expression is reported to be strongly affected by inflammatory cytokines such as TNF-α, interleukin (IL)-1α, IFN-γ, and TGF-β and hypoxia-induced mechanisms involving hypoxia-inducible factors. Furthermore, CSPG4 expression was described to depend on epigenetic pathways, certain transcription factors and microRNAs (see Ampofo et al. for review).

**Figure 1:**
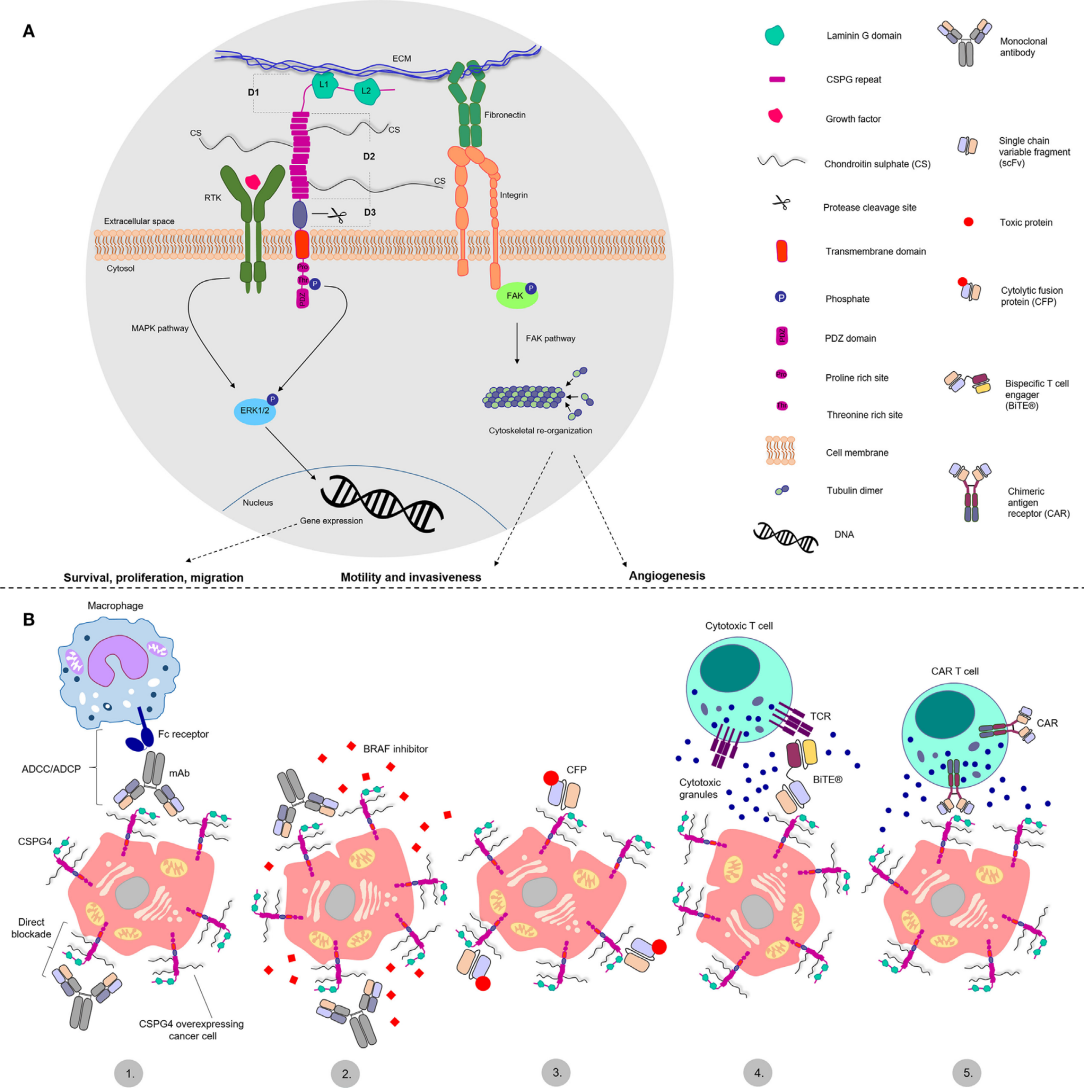
Structure and functions of chondroitin sulfate proteoglycan 4 (CSPG4) and antibody-based treatment approaches. **(A)** Schematic representation of CSPG4 proposed structure and functions in cancer. CSPG4 has three extracellular domains: D1, D2 and D3. Domain 1 (D1) consists of two laminin G like domains (L1 and L2) proposed to interact with the extracellular matrix (ECM). Domain 2 (D2) consists of 15 CSPG repeats containing chondroitin sulfate chain decoration. It is proposed to interact with integrins and ECM proteins, and to bind and present growth factors to receptor tyrosine kinases. Domain 3 (D3) contains putative protease cleaving sites and may be involved in protein shedding. The cytoplasmic tail containing proline- and threonine-rich sites, is thought to interact with different proteins and function as a phosphoacceptor site for the extracellular signal-regulated kinase 1/2 (ERK1/2), respectively. The PDZ domain is involved in protein scaffolding functions. CSPG4 is therefore implicated in cellular signaling pathways, including the mitogen-activated protein kinase pathway, through the receptor tyrosine kinase-ERK1/2 axis and the focal adhesion kinase (FAK) pathway, through the ECM–fibronectin–integrin axis. These may promote survival, proliferation and migration, cytoskeletal reorganization that may promote motility, invasiveness, and angiogenesis. **(B)** Key cancer antibody immunotherapy strategies targeting CSPG4: 1. Classic antibody approaches, functioning through two mechanisms—direct blockade of cell signaling functions and antibody dependent cellular cytotoxicity/phagocytosis (ADCC/ADCP) mediated by immune effector cells like macrophages and NK cells; 2. Combination of CSPG4 blocking antibodies and BRAF inhibitors; 3. Cytolytic fusion proteins (CFPs); 4. Bispecific T cell engager antibodies (BiTEs) redirecting cytotoxic T cells toward CSPG4 overexpressing cells; 5. Chimeric antigen receptor (CAR) T cells, redirecting genetically modified T cells toward CSPG4 overexpressing cells.

Its functional versatility could be explained by its protein scaffold structural characteristics (Figure 1). CSPG4 is a type I single pass transmembrane protein which exists as a core glycoprotein and chondroitin sulfate-decorated proteoglycan (18). Studies with the rat ortholog state CSPG4 consists of a large extracellular portion, a transmembrane domain and a short intracellular portion (19). The extracellular portion comprises three distinct domains. Located furthest from the membrane, D1 is composed of two laminin G-type subdomains and is abundant in disulfide bonds, important for the stability of tertiary structure. This domain is potentially involved in the interactions with the extracellular matrix (20). The middle domain, D2, comprises 15 CSPG4 specific repeats containing several potential glycosylation and chondroitin sulfate binding sites. The CS decoration may confer different attributes, including interaction with integrins and metalloprotease activation (21, 22). It is presently unclear whether CSPG4 is characterized with different glycosylation/glycanation patterns in normal or cancerous tissues. The D2 domain has also been proposed to directly bind collagens (23, 24). Although CSPG4 has no reported enzymatic functions, murine ortholog studies suggest it may bind growth factors and present them to receptor tyrosine kinases (RTKs), thus acting as a RTK coreceptor (25, 26).

Domain D3 is the one proximal to the cellular membrane, and contains putative protease cleavage sites as well as carbohydrate decoration, suggesting potential interactions with lectins and integrins (27, 28). Proteolytic cleavage may also allow ectodomain shedding. In support, levels of soluble CSPG4 have been reported in the sera of healthy individuals and patients with melanoma (29). The presence of soluble CSPG4 within circulation has been proposed as a potential diagnostic biomarker to aid melanoma detection and classification at the vertical growth phase (29). Moreover, CSPG4 may undergo endocytic recycling mediated by the endocytic receptor Stonin1 (30). Thus, endocytosis and ectodomain shedding of CSPG4 may point to different mechanisms involved in the turnover of membrane-bound protein.

The intracellular portion of CSPG4 is characterized by the presence of a threonine- and a proline-rich motif and a PDZ [postsynaptic density protein 95 (PSD-95)—*Drosophila* disc large tumor suppressor (Dlg1)—Zona occludens 1 (ZO-1)] domain (31, 32). The threonine motifs serve as kinase phosphoacceptor sites for protein kinase C α (PKCα) and extracellular signal-regulated kinase 1/2 (ERK1/2) (33, 34). The proline-rich domain and the PDZ domain most likely function as protein scaffolds for other intracellular proteins (31). The structural characteristics of CSPG4 may confer possible functions as a signaling mediator molecule, connecting the extracellular matrix (ECM) with two main intracellular signaling cascades—the integrin-focal adhesion kinase (FAK) axis through integrin interactions and the mitogen-activated protein kinase (MAPK) pathway through activation of RTKs and ERK1/2 (33, 35, 36). These may bestow functional attributes that could encompass promotion of cellular survival, proliferation, and motility. Importantly, studies with NG2 knockout mice suggest that deletion of this antigen is not lethal (37).

Therefore, research studies into CSPG4 point to stem cell origin, multiple functions in tissue development, together with regulation of expression by inflammation, hypoxia, decreased methylation mechanisms, as well as specific protein scaffolding structure and reported roles in cell signaling. These features may also translate to contributions in cancer pathology in several tumor types.

## CSPG4 Expression and Putative Pathological Functions in Cancer

Early reports associated overexpression of CSPG4 with malignant melanomas, and more recently its enhanced expression has been identified in other cancer types. Despite these developments, the exact involvement of CSPG4 in the etiology of cancer is widely unknown.

It remains unclear whether CSPG4 has a role in tumor initiation or its expression only accumulates in tumors as a secondary tumor-associated event. Overexpression of CSPG4 has not been reported to be a result of genetic aberrations such as gene amplifications or chromosome translocation, suggesting CSPG4 may not be a primary driver in the onset of cancer. Even though its expression is not connected to the onset of epithelial tumors or hematological cancers, it has been linked to the putative mesenchymal stem cell origin of sarcomas and the epithelial–mesenchymal transition in melanoma, thought to be important for malignant transformation (36, 38–40). Several reports point toward potential roles in cancer growth and dissemination. Expression of the CSPG4 rodent homolog has been shown to localize in the invasive front of the filopodia of oligodendrocytes, suggesting involvement in mediating tumor cell motility and cancer dissemination (38, 41). In support, immunohistochemistry data evaluating CSPG4 expression in human melanoma claim higher levels in metastatic lesions than in primary tumors, and CSPG4 mRNA expression was reported to be predictive of metastasis formation in soft tissue sarcoma patients (6, 42).

Furthermore, CSPG4 is believed to contribute to cancer growth and progression through promotion of angiogenesis. Using cell line and patient derived glioblastoma xenografts in nude mice, Wang et al. reported that NG2 RNA interference *in vivo* decreased tumor volume and vasculature (43). In addition, a retrospective study focused on germline polymorphisms related to the function of pericytes in colorectal cancer, identified a CSPG4 polymorphism to be predictive of lower progression-free survival in patients treated with the monoclonal antibody (mAb) bevacizumab—specific for the vascular endothelial growth factor (44). More evidence is required to clarify the exact mechanism through which CSPG4 promotes angiogenesis in cancer.

Disparate reports therefore point to potential contributions of CSPG4 in cancer growth, vascularization, dissemination, and metastasis (Figure 1). These may provide opportunities for therapeutic interventions targeting CSPG4 and multiple cancer-associated pathways this molecule may be involved in.

### Expression in Neuroectodermal Cancers

#### Malignant Melanoma

Among the neuroectodermal tumors, malignant melanoma is the most thoroughly characterized in terms of CSPG4 expression and functions (36). CSPG4 is the only well characterized cell surface melanoma-associated antigen and it has been examined as a potential target employing different therapeutic approaches. It is expressed in over 70% of melanomas, and its expression has been detected throughout different disease stages.

Multiple studies demonstrate the importance of a functional full length CSPG4 for the survival, growth, and motility of melanoma tumor cells *in vitro* and tumor formation *in vivo*. CSPG4 contributes to enhanced activation of the integrin-FAK pathway through interaction with ECM components, which leads to integrin clustering and subsequent downstream signaling, cytoskeletal reorganization and increased motility and invasiveness (14, 21, 27, 33, 35). On the other hand, CSPG4 facilitates the MAPK pathway through activation of ERK1 and ERK2. ERK1 and 2 can then regulate the microphthalmia-associated transcription factor, which then alters the levels of vital proteins—increasing the expression of fibronectin, but also repressing the expression of E-cadherin, both of which have been shown to be associated with metastasis (45–49).

Since a big proportion of melanomas feature constitutively active MAPK pathway due to mutations in the gene encoding the serine/threonine-protein kinase, BRAF, several groups have investigated the effect of CSPG4 in BRAF mutant melanoma *in vitro*. Full length CSPG4 is required for maximal activation of ERK1/2, and siRNA inhibition of CSPG4 leads to a reduction in ERK signaling (50). Importantly, *in vitro* studies report that specific small molecule inhibitors for mutant BRAF can synergize with the 225.28 anti-CSPG4 mAb in inhibiting cell growth and proliferation (51). On the other hand, ERK1/2 signaling blockade leads to reduced CSPG4-dependent cancer cell motility (50). These reports suggest that alongside constitutive activation of BRAF, CSPG4 may additionally promote MAPK pathway activation.

#### Glioma

Expression of CSPG4 is found in subsets of normal glial cells in developing and adult central nervous system (see Dimou and Gallo for a review) (52). Although less well examined than melanoma, CSPG4 expression has also been associated with gliomas that originate from astrocytes, such as glioblastoma multiforme (GBM) (53–55). In a study analyzing mRNA expression data sourced from The Cancer Genome Atlas, CSPG4 mRNA levels were reported to be elevated compared to normal tissue controls (54). As in melanoma, functions of CSPG4 in glioblastoma are believed to be related to malignant progression through facilitating tumor cell interactions with collagen and promoting angiogenesis (56, 57). Interestingly, as reviewed by Ampofo et al., the expression levels of both CSPG4 and the platelet-derived growth factor receptor alpha (PDGFR-α) in glioma are downregulated by micro RNA miR192-2 (17). Moreover, CSPG4 and PDGFR-α have been reported to interact and enhance cell proliferation upon PDGF stimulation. Therefore, miR129-2 has been proposed by Ampofo et al., to have therapeutic potential in glioma. CSPG4 has also been associated with pediatric brain tumors such as medulloblastoma (58–60).

### Expression in Epithelial Cancers

#### Breast Cancer

Another cancer type of epithelial origin, whose associations with CSPG4 have attracted a surge of interest, is breast cancer. Expression of CSPG4 in triple-negative breast cancer (TNBC) in particular is of special value, since this subtype represents an area of unmet need for novel targeted therapies. TNBCs, comprising 15% of all breast cancers, lack expression of the estrogen, progesterone and the HER2. Their triple-negative status makes them insensitive to the current hormone and HER2 targeted therapies. TNBCs are more aggressive with worse prognosis compared with other breast cancer types with no targeted therapies available, and therefore, novel targets and treatment options are urgently required.

Chondroitin sulfate proteoglycan 4 expression was described in TNBC primary lesions and metastatic tumor cells from pleural infusions, including cancer stem cells (61). Although CSPG4 expression does not appear to be expressed exclusively by basal breast cancers including TNBCs, its expression may be associated with poor prognosis and relapse in breast cancers (62). Findings to-date indicate a link between the expressions of carbohydrate sulfotransferase-11 (encoded by CHST11) which is involved in decorating CSPG4 with chondroitin sulfate and the metastatic behavior of triple-negative breast cancer cells (63). Reportedly, CHST11 is overexpressed in aggressive breast cancers and facilitates the interaction between p-selectin and CSPG4. These observations are in line with the proposed role of CSPG4 as a mediator between the ECM and intracellular signaling pathways and a metastatic driver, P-selectin. The latter is thought to allow cancer cells to resist the immune response, and to support binding to endothelial cells, and thus hematogenous spread (64). Further research is required to elucidate the CSPG4 pathological contributions to breast cancer formation and progression.

#### Head and Neck Cancers

Head and neck squamous-cell carcinomas (HNSCC) normally have a poor prognosis, with a 5-year survival rate of around 40–50%. Warta et al. recently reported CSPG4 to be significantly overexpressed in HNSCC cells (65). Furthermore, high expression in patient lesions was found to correlate with poorer prognosis compared with low expression of CSPG4 (65). The study noted that few biomarkers are currently available to predict survival in HNSCC, and that CSPG4 could in future serve as a prognostic indicator.

### Expression in Mesenchymal Cancers

A recent study using murine sarcoma models demonstrated that both malignant bone and soft tissue sarcomas, as well as benign desmoid tumors could originate from CSPG4-expressing pericyte cells (66). Nevertheless, the authors did not investigate the specific implications of CSPG4 expression in the tumor formation process. Another potential mechanism described by Cattaruzza et al. suggests an interplay between CSPG4 and type VI collagen in the progression of soft-tissue sarcoma (67). The same study reports upregulation of both CSPG4 and collagen type VI in soft-tissue sarcoma lesions and suggests that the combination can predict metastasis and poor clinical prognosis. However, the role of CSPG4 in mesenchymal tumors is yet to be revealed.

### Expression in Hematological Cancers

Chondroitin sulfate proteoglycan 4 expression has also been reported in acute myeloid leukemia and acute lymphoblastic leukemia (ALL), while it is not expressed on normal lymphocytes, granulocytes or hematopoietic progenitor cells (68–71). Remarkably, for both ALL and AML, CSPG4 expression strongly correlated with the 11q23 gene rearrangement of the KMT2A gene encoding the lysine methyltransferase 2A. The mechanisms behind this correlation are still unknown. Nicolosi et al., shared an unpublished observation that a potential function of CSPG4 in leukemic malignancies could be induction of drug resistance, based on CSPG4 *in vitro* knock-in experiments in mixed lineage leukemia gene bearing cells, resulting in increased drug transporter expression (38).

## CSPG4 and Cross-Talk with the Immune System

Several studies have investigated the interactions between the chondroitin sulfate chains of proteoglycans or their degradation products and components of the immune system.

It has been reported that CS could stimulate monocytes to secrete IL-1β and to induce B cell proliferation *in vitro* (72). The effect of CS on B cell proliferation was corroborated by Aoyama et al., who demonstrated that CS could enhance murine B cell proliferation *in vitro* through PKC translocation and activation of protein kinase B (PKB, Akt) kinase (73). A role of CS in the maturation of dendritic cells was suggested with human monocyte-derived dendritic cells cultured *in vitro* in the presence of CS, hyaluronic acid, components of the ECM and human granulocyte-macrophage colony-stimulating factor (GM-CSF). CS-stimulated cells could differentiate faster than when cultured with GM-CSF and IL-4 (74). Another study utilizing splenocytes from ovalbumin (OVA)-immunized mice cultured *ex vivo*, showed that CS addition to culture stimulated secretion of Th1-type cytokines including IFN-γ, IL-2, and IL-12 and suppressed the secretion of Th2-type cytokines (IL-5 and IL-10) (75). Moreover, injection of BALB/c mice with CS and other glycosaminoglycans (GAGs) was shown to induce autoimmune conditions like rheumatoid arthritis through recruitment of CD4^+^ T cells (76). In addition, treatment of murine NK cells with chondroitinase or a proteoglycan biosynthesis inhibitor resulted in substantial decrease in IFN-γ secretion through interaction with murine IL-12. On the other hand, CSPG low-molecular weight disaccharide fragments could control the inflammatory response in a mouse model of autoimmune encephalitis, in a rat model of inflammation-mediated neuropathology of the eye, and in a delayed-type hypersensitivity model in Balb/c mice through reduced migration and activation of inflammatory T cells (77).

With regards to the role of chondroitin sulfate proteoglycans (CSPGs), an early study reported these located inside the granules of human NK cells and their exocytosis during NK cell-mediated cytotoxicity of tumor cells (78). Furthermore, a study of primary cultured human macrophages, showed secreted CSPGs as metabolic products of macrophages and increased secreted CSPG4 following lipopolysaccharide (79) stimulation (80).

A more recent immune monitoring study identified that both healthy individuals and melanoma patients have circulating CSPG4-reactive CD4^+^ T cells. The study did not report a significant correlation between the T cell responses against a HLA-DR presented CSPG4 peptide, quantified through IFN-γ production, and the tumor burden of patients. The authors showed that a smaller proportion of the melanoma patients (11 out of 42) compared to healthy volunteers (11 out of 13) exhibited T cell reactivity to CSPG4 (81). Other *in vivo* studies demonstrated that LPS induced the expression of the murine CSPG4 ortholog by rat microglia cells. Further *in vitro* experiments showed that NG2 RNA silencing of LPS-treated microglia blocked the mRNA expression of nitric oxide synthase (Yajima et al.), and of proinflammatory cytokines including IL-1β and tumor necrosis factor α (TNF-α), but not of chemokines like monocyte chemoattractant protein 1 and stromal cell-derived factor 1 α (SDF-1α). Importantly, this study demonstrated that NG2 is not only expressed upon stimulation of the microglia, but it likely has a role in regulating expression of pro-inflammatory cytokines (82).

In summary, chondroitin sulfate proteoglycans (CSPGs), CS carbohydrate chains, as well as small molecular weight CS degradation products and CSPG4, each appear to influence the activation, maturation, proliferation and migration of different immune cell subsets. The definitive roles for CSPG4 in the immunology of cancer are however still widely unexplored. More research is required to clarify whether the interactions between CSPG4, its amino acid or carbohydrate moieties and different components of the immune system could be exploited to enhance patient response to CSPG4 targeted immunotherapy or to be counteracted to avoid potential negative immunomodulatory functions.

## CSPG4 as a Target for Antibody Therapies in Cancer

Since CSPG4 is found to be overexpressed in a number of malignancies and based on its low and restricted distribution in normal tissues, alongside emerging evidence for crucial roles in cancer growth and dissemination, much research has focused on the development of different therapeutic approaches, including monoclonal antibodies. Some of these antibody-based strategies focused on CSPG4 are discussed below.

### Classic mAb Approaches

Published studies describe a limited number of mAb clones recognizing CSPG4, the most commonly cited of which is a murine clone, 225.28. One of the earliest efficacy studies reports the antitumor efficacy of the murine 225.28 conjugated to methotrexate in nude mice bearing human melanoma xenografts (83). Even though conjugated mAb had superior efficacy to methotrexate alone, the efficacy of the mAb alone was not investigated. Melanoma tumor-bearing SCID mice treated with the murine mAb 225.28 bore smaller tumors compared with controls, and treatments were associated with modulation of various tumor suppressor-genes and genes involved in cancer metastasis (84). Besides melanoma, this murine mAb was also reported to inhibit the proliferation, adhesion and migration of TNBC cells, as well as to downregulate tumor-promoting signaling pathways *in vitro*. Moreover, the murine mAb was demonstrated to reduce tumor growth in two human TNBC cell line-derived lung metastasis models in SCID mice, and to decrease tumor angiogenesis and tumor recurrence after surgical removal in an orthotopic human TNBC cell line xenograft model in SCID mice (61). The murine antibody clone 225.28 was later also demonstrated to restrict tumor growth synergistically in combination with BRAF inhibitors *in vitro* (51). The anticancer potential of mAb 225.28 was tested against 11q23 ALL cells (71). The mAb on its own showed no direct effects on proliferation inhibition in ALL cells *in vitro*, but it increased the efficacy of the chemotherapy agent Cytarabine. The mAb 225.28 also showed some tumor growth restriction efficacy in a subcutaneous ALL model in SCID mice. One of the first studies reporting an antibody engineered with a human Fc region, was of a chimeric version of the mouse mAb 225.28. The study showed that this mAb could mediate antibody-dependent cellular phagocytosis (ADCP) by human monocytes *in vitro* and could restrict tumor growth *in vivo* in a melanoma NOD-SCID-Gamma (NSG) mouse model engrafted with human immune cells (85) Interestingly, anti-CSPG4 IgG4 was demonstrated to not only lack tumor inhibition properties *in vivo*, but to also impair the efficacy of its IgG1 analog when administered in combination. This finding highlights the importance of choosing appropriate Fc domain and antibody isotype when designing therapeutic mAbs. Moreover, the original murine anti-CSPG4 clone 225.28 has been reported to exhibit direct cancer cell proliferation inhibition properties. In this context, the data reported by Karagiannis et al. opens different avenues for discussion and future investigation, concerning the effect of chimerizing or humanizing mAbs on their direct blockade properties, as well as the magnitude of the immunologically induced anticancer effects engendered by mAb immunotherapy.

Chondroitin sulfate proteoglycan 4 has also been targeted in other malignant indications using different antibody clones. The murine mAb clone TP41.2 was used in malignant mesothelioma (86). TP41.2 showed *in vitro* antitumor effects by reducing cancer cell proliferation, adhesion, motility, migration and invasion. The antibody also reduced tumor growth *in vivo* and prolonged the survival of mesothelioma-bearing SCID mice. In a different study by Wang et al. the authors utilize a single-chain variable fragments (scFv) fused to a human IgG1 Fc portion—scFv-FcC21 and demonstrated growth and migration inhibition of a TNBC cell line *in vitro*, as well as reduction of lung metastasis in a melanoma cell line model in SCID mice *in vivo* (87). The scFc-FcC21 antibody was shown not to be able to induce antibody-dependent cellular cytotoxicity or phagocytosis *in vitro*. Furthermore, an antimouse CSPG4/NG2 antibody clone 9.2.27 was conjugated to polyethylene glycol to increase its affinity for rat FcγRIII on rat NK cells, and was used in combination with adoptive NK cells in glioblastoma engrafted athymic rats. The combination of adoptive NK cell transfer and PEGylated mAb 9.2.27 could restrict tumor growth *in vivo* (88). Curiously, the authors accounted the therapeutic efficacy to macrophages recruited to the tumor, whose clodronate-mediated depletion abolished the antitumor effects of the mAb. The authors suggested that the adoptively transferred NK cell plus mAb treatment might be responsible for re-educating tumor-infiltrating macrophages to render antitumor functions, however, the exact mechanism remains unknown. Interestingly, the antigenic determinants of the monoclonal antibodies, described in the above section, have been suggested to recognize the CSPG4 core protein independently of the presence of chondroitin sulphate (89, 90). It has also been proposed that removal of the CS decoration from the core CSPG4 protein would not affect the reactivity towards CSPG4 of any of the murine clones, mentioned above. Nevertheless, these observations require further confirmation (89).

Even though certain anti-CSPG4 antibody clones showed promise for therapeutic application in different cancer types and in a number of *in vitro* and *in vivo* models, most of published studies were performed using antibodies with mouse Fc regions. With some encouraging findings against CSPG4-expressing tumors, the potential of anti-CSPG4 antibodies against cancer would benefit from further in-depth research using novel constructs including those engineered with human Fc regions.

### Anti-idiotypic Antibodies

Early strategies targeting CSPG4 utilized the development of anti-idiotypic antibodies (anti-id), which target the binding sites of other anti-CSPG4 antibodies, essentially mimicking the tumor antigen’s binding site on the antibody, and thus aiming to serve as immunogens or vaccines (91, 92). A clinical study reported increased survival and metastasis regression in patients with melanoma who developed anti-CSPG4 antibodies following administration of the anti-idiotypic mAb MK2-23 (93). Despite the promising clinical results, MK2-23 never reached clinical application as a therapy due to standardization issues and safety concerns linked to its administration together with the adjuvant Bacille Calmette-Guerin (BCG), deemed necessary for triggering effective adaptive immune responses (94). Further approaches to overcome those issues included fusing MK2-23 to human IL-2 (94) or utilizing DNA vaccines encoding MK2-23 scFv (95). In a phase I clinical trial, another anti-idiotypic antibody, MF11-30, induced complete remission in one advanced melanoma patient as well as conferred minor survival benefits in three other patients with advanced melanoma whilst no toxicities were reported as a part of Ref. (96).

Another approach concerns vaccine approaches, such as mimotope vaccination studies utilizing conformational CSPG4 epitopes recognized by the 763.74 or the 225.28S anti-CSPG4 mAb clones. Such strategies have been studied and reported induction of CSPG4-specific antibodies in mimotope-vaccinated animals, as well as promising antimelanoma activity of these antibodies *in vitro* through direct proliferation inhibition or in murine effector cell ADCC assays (97–99).

Even though anti-id antibody strategies or mimotope vaccines are currently not in the spotlight, these early studies indicate that CSPG4 may be a promising target for vaccine-based cancer immunotherapy.

### Bispecific T Cell Engagers

Bispecific T cell engagers represent a novel therapeutic modality based on the fusion of two single-chain variable fragments (scFv), one of which binds the target antigen while the other engages with T cells *via* CD3. Unlike classic monoclonal antibodies, BiTEs are designed to activate cytotoxic T cells against tumor cells. In 2011, a new CSPG4-targeting BiTE antibody was shown to induce antitumor effects *in vitro via* redirected lysis (100). Following incubation with healthy donor PBMCs, all of the 23 melanoma cell lines utilized in the study were successfully lysed in a dose- and effector:target ratio-dependent manner. Furthermore, the BiTE antibody showed promise in a melanoma patient setting *in vitro* by triggering cytotoxicity by melanoma patient-derived T cells against allogeneic or autologous melanoma cells. Another study reports important findings about the design of CSPG4-targeting BiTE therapeutics linked to the epitope distance to the target cell (101). Anti-CSPG4 BiTE antibodies proved much more potent when binding epitopes located closer to the cell membrane. This was proposed to be linked to the large size of the CSPG4 antigen and represents an important factor to consider for the design of antibody therapeutic agents with maximal potency.

### CAR T Cells

Chimeric Antigen Receptor T cells represent another promising T cell-based therapeutic approach utilizing monoclonal antibodies whose efficacy against CSPG4 is being investigated. CAR T cells are genetically modified to express a chimeric receptor based on the targeting moiety of a mAb (scFv) recognizing the antigen of interest, to re-direct cytotoxic T cells toward tumor cells. The robust clinical success in the treatment of ALL has attracted a lot of attention on CAR T cell approaches, with one anti-CD19 based therapy expected to soon be granted FDA approval for the treatment of pediatric ALL (102). One of the first studies investigating the potential of anti-CSPG4 CAR T cells showed *in vitro* cytolytic potency against a variety of solid tumor cell lines including breast cancer, melanoma, mesothelioma, glioblastoma and osteosarcoma (103). Another study investigating anti-CSPG4 CAR T cells announced promising efficacy outcomes against melanoma, breast cancer and head and neck cancer *in vitro* and *in vivo* using cell line xenografts in mice (104).

Even though preclinical data on anti-CSPG4 CAR T cell therapy are encouraging, it remains to be established whether the clinical responses observed with CAR T cell treatments against liquid tumors can be reproduced in solid malignancies. A prominent limitation of the efficacious re-targeting of T cells against solid tumors is the tumor stroma, often inhibiting T cell trafficking and potency. Multiple approaches have been designed to address this issue, including the expression of chemokine receptors by the CAR T cells or *in vivo* tumor modification to encourage the secretion of T cell chemoattractants (105).

### Radioimmunotherapy

Monoclonal antibodies recognizing CSPG4 could be conjugated to a radioactive isotope for radioimmunotherapy, designed to target radiation directly and more specifically to tumor cells, with the aim of reducing non-specific exposure of normal cells to the radioactive isotope. Targeted radioimmunotherapy may be promising with regards to CSPG4, based on overexpression in advanced tumors, restricted distribution and lower levels of expression in normal tissues (106). So far, radioimmunotherapy has only been successfully applied for the treatment of patients with lymphoma, while most clinical trials in solid tumors have rarely reached phase III, often due to cost, or to stringent patient inclusion criteria (107). Nevertheless, the outcomes of a phase I clinical trial of (213)Bi-cDTPA-9.2.27 (based on the anti-CSPG4 mAb clone 9.2.27) in advanced melanoma indicated no toxicities, a 10% objective partial response rate (108). Furthermore, a more recent preclinical study evaluating mAb 225.28 radiolabeled with 212Pb showed efficacy against triple negative breast cancer cells expressing CSPG4, both *in vivo* and *in vitro* (109).

Based on these findings, radioactive isotope-conjugated anti-CSPG4 antibodies may yet hold promise for patients with CSPG4-expressing tumors.

### Cytolytic Fusion Proteins (CFPs)

Cytolytic fusion proteins or immunotoxins, are classified as protein toxins, most commonly of plant or bacterial origin, genetically fused or conjugated to another protein (often an antibody or an antibody fragment), recognizing a cell surface target molecule and delivering the payload to the cancer cell (110).

The microtubule-associated protein tau, recently investigated as a toxin, primarily functions as a microtubule stabilizer and a regulator of cell division (111). Targeting CSPG4 using a scFv fused to MAP against TNBC cell lines *in vitro* and against human TNBC cell line xenografts in Balb/c mice showed similar antitumor efficacy to an anti-CSPG4 scFv conjugated to a chemotherapeutic agent, with no toxic effects *in vivo* (112).

Another interesting approach involves an anti-CSPG4 scFv conjugated to the TNF-related apoptosis-inducing ligand (TRAIL), a soluble protein ligand able to induce apoptosis through binding the cell surface-anchored TRAIL receptor. In a study investigating a TRAIL-fused anti-CSPG4 scFv (based on the mAb 9.2.27), the novel therapeutic candidate showed potent *in vitro* activity against melanoma cell lines, but no off-target effects on normal melanocytes (113, 114). Moreover, it restricted the growth of a human melanoma xenograft in athymic mice.

Both CFP examples suggest that this may represent a promising targeted delivery alternative to chemotherapy, especially for antigens such as CSPG4 with high expression in tumors and restricted expression in normal tissues.

## Combination Therapies and Other Applications

BRAF inhibitors, now approved for the treatment of patients with melanomas bearing mutant forms of BRAF, are often only effective for a short time before cancer recurs, due to intrinsic and acquired pathway resistance (115). Therefore, alternative treatments and treatment combinations that may overcome resistance mechanisms are desirable (116). The anti-CSPG4 mAb 225.28 combined with a BRAF inhibitor exhibited synergistic antitumor effects and enhanced efficacy against BRAF^V600E^ mutant melanoma cells *in vitro* compared to either agent alone (51). Furthermore, the mAb was shown to delay the development of resistance by melanoma cells. More recently, Pucciarelli et al. showed that combining polyclonal anti-CSPG4 antibodies, induced by mimotope vaccination, with the BRAF inhibitor vemurafenib synergistically reduced the proliferation and migration of melanoma cells *in vitro* (117). These preliminary findings suggest that combining anti-CSPG4 antibodies with pathway inhibitors may enhance the restricted success of BRAF inhibitors in melanoma.

Cancer theranostic agents, combining both diagnostic and therapeutic treatment in one targeted molecule are an emerging modality. As with other antibody therapeutic applications, CSPG4 has been recognized as a promising candidate for theranostic applications, based on high expression by tumor cells and low expression by healthy cells. In an *in vitro* photo-immunotheranostic study, single-chain variable fragment (scFv) antibodies recognizing TNBC targets, including CSPG4, as well as epidermal growth factor receptor (EGFR) and epithelial cell adhesion molecule (EpCAM), were conjugated to a potent photosensitizing agent and were used to target TNBC cell lines and tumor biopsy samples (118). The conjugated scFvs demonstrated high quality imaging capacity, and triggered apoptosis of cancer cells *via* induction of reactive oxygen species. Moreover, combinatorial administration of all three conjugated scFv antibodies together, increased cytotoxic activity against breast cancer cells *in vitro* by up to 40% compared with treatment by each individual agent alone. Further findings are awaited to confirm and provide further efficacy insights on these encouraging outcomes in future *in vivo* studies.

## Conclusion and Future Directions

Despite advances in immunotherapy such as the emergence of checkpoint inhibitors for melanoma, mAb, or CAR T cell strategies that specifically target melanoma cells are still lacking. Localized overexpression in several aggressive tumor types and in tumor vasculature, combined with low and restricted distribution in normal tissues, as well as evidence for important functions to support cancer growth, angiogenesis and dissemination, represent important attributes that identify CSPG4 as a promising target for therapeutic approaches, including monoclonal antibodies (Table 1). Importantly, in order to develop more successful therapeutics, a better understanding of the functions of CSPG4 in cancer and its interaction with the immune system and the tumor immune stroma are urgently needed.

**Table 1:** Antibody-based treatment approaches targeting CSPG4.

Treatment strategy	Clone/construct	Toxin conjugate	Treatment combination	Antibody species	*In vitro* model and indication	*In vivo* model and indication	Proposed mechanism(s) of action	Clinical trial	Key reference
Classic mAb	225.28S	MTX	N/A	Full mouse antibody	Melanoma cell line	Human melanoma cell line xenograft; nude mice	Growth inhibition, delivery of cytotoxic drugs to the tumor	N/A	(83)
Classic mAb	225.28S	N/A	N/A	Full mouse antibody	Melanoma, TNBC cell line	Human melanoma cell line xenograft; SCID mice	Disruption of the interaction between the cancer cells and the ECM	N/A	(84)
Classic mAb	225.28S	N/A	N/A	Full mouse antibody	TNBC cell line	Human TNBC cell line lung metastasis model; SCID mice Orthotopic human TNBC cell line xenograft; SCID mice	Direct effects (growth, adhesion, and migration inhibition)	N/A	(61)
Classic mAb	225.28S	N/A	PLX4032 (BRAF inhibitor)	Full mouse antibody	Human BRAF^V600E^ mutant melanoma cell lines	N/A	Synergistic direct effects (growth, migration, survival inhibition); delayed BRAF inhibitor resistance	N/A	(51)
Classic mAb	225.28S	N/A	Cytarbine (chemotherapy)	Full mouse antibody	11q23 AML cell line	Human AML cell line subcutaneous model; SCID mice	No direct effects (mAb alone); synergistic antiproliferative effects with Cytarbine; no *in vivo* effects on tumor growth/animal survival	N/A	(71)
Classic mAb	225.28S	N/A	N/A	Chimeric antibody (mouse Fab, human Fc)	Melanoma cell lines	Human melanoma cell line xenograft; human immune cell engrafted NSG mice	Immune mediated effects (ADCC/ADCP)	N/A	(85)
Classic mAb	TP41.2	N/A	N/A	Full mouse antibody	Mesothelioma cell line	Human mesothelioma cell line xenograft; SCID mice	Direct effects (cell growth, adhesion, motility, migration, and invasiveness)	N/A	(86)
Classic mAb	scFv-FcC21	N/A	N/A	Recombinant scFv mAb with a human Fc region	TNBC cell line	Human melanoma cell line xenograft; SCID mice	No immune mediated effects; direct effects (cell growth and migration inhibition)	N/A	(43, 87)
Classic mAb	9.2.27	PEG	Adoptive NK cell transfer	Full mouse antibody	N/A	Human GBM cell line xenograft, patient derived GBM xenograft; athymic rats	Immune-mediated effects by macrophages	N/A	(88)
Classic mAb	Polyclonal mAbs	N/A	Vemurafenib (BRAF^V600E^ inhibitor)	Full mouse antibody	Human BRAF^V600E^ mutant melanoma cell lines	N/A	Synergistic direct effects (proliferation and migration inhibition)	N/A	(117)
Anti-idiotypic mAb	MK2-23	N/A	BCG	Full mouse antibody	N/A	N/A	Induction of adaptive humoral immune response	Advanced melanoma patients; Phase I/II	(93)
Anti-idiotypic mAb	MK2-23	IL-2	N/A	Full mouse antibody	N/A	BALB/c mice	Induction of adaptive humoral immune response	N/A	(94)
Anti-idiotypic mAb	MF11-30	N/A	N/A	Full mouse antibody	N/A	N/A	Induction of adaptive humoral immune response	Advanced melanoma patients; 2× phase I	(96)
BiTE	MCSP-BiTE	N/A	N/A	Recombinant BiTE construct	Human melanoma cell lines; melanoma patient-derived samples	N/A	Cytotoxic T cell-mediated tumor cell killing	N/A	(100)
BiTE	MCSP120, MCSP128, MCSP113, MCSP70	N/A	N/A	Recombinant BiTE construct based on mouse hybridoma-derived mAbs	CHO cells expressing CSPG4 domain portions	N/A	Cytotoxic T cell-mediated tumor cell killing	N/A	(101)
CAR	CAR constructs based on mAbs 225.28; TP41.2; 149.53	N/A	N/A	Recombinant CAR construct, includes mAb scFv region based on mouse mAbs	Human melanoma, breast cancer, mesothelioma, glioblastoma and osteosarcoma cell lines	N/A	Cytotoxic CAR T cell-mediated tumor cell killing	N/A	(103)
CAR	CAR construct based on mAb 763.74	N/A	N/A	Recombinant CAR construct, includes mAb scFv region based on mouse mAbs	Human, melanoma, HNSCC and breast cancer cell lines	Human, melanoma, HNSCC and breast cancer cell line xenografts; NSG mice	Cytotoxic CAR T cell-mediated tumor cell killing	N/A	(104)
CAR	CAR construct based on mAb 225.28	N/A	N/A	Recombinant CAR construct, includes mAb scFv region based on mouse mAbs	Human melanoma cell lines	N/A	Cytotoxic CAR T cell-mediated tumor cell killing	N/A	(119)
Radioimmunotherapy	(213)Bi-cDTPA-9.2.27 (based on mAb 9.2.27)	(213)Bi-cDTPA	N/A	Full mouse antibody	N/A	N/A	Radiotherapy induced targeted cytotoxicity	Advanced melanoma patients, phase I	(108)
Radioimmunotherapy	225.28S	212Pb	N/A	Full mouse antibody	Human TNBC cell line	Human TNBC cell line xenograft; nude mice	Radiotherapy induced targeted cytotoxicity	N/A	(109)
CFP	αCSPG4(scFv)-MAP	MAP	N/A	Recombinant construct, scFv mAb genetically fused to MAP	Human TNBC cell line	Human TNBC cell line xenograft; BALB/c mice	MAP induced targeted cytotoxicity	N/A	(112)
CFP	Anti-MCSP TRAIL (based on mAb 9.2.27)	TRAIL	N/A	Fully mouse mAb genetically fused to TRAIL	Human melanoma cell lines	Human melanoma cell line xenograft, athymic mice	TRAIL induced targeted cytotoxicity	N/A	(114)
Photoimmunotheranostics	Anti-CSPG4 (scFv)-SNAP-tag	IR700 (photosensitizing agent)	Anti-EGFR (scFv)-SNAP-tag and anti-EpCAM(scFv)-SNAP-tag	Recombinant construct, scFv mAb genetically fused to SNAP tag	Human TNBC cell lines	N/A	Phototherapeutic activity	N/A	(118)

*mAb, monoclonal antibody; MTX, methotrexate; PEG, polyethylene glycol; TNBC, triple-negative breast cancer; SCID, severe combined immunodeficiency; ECM, extracellular matrix; NSG, NOD scid gamma; GBM, glioblastoma multiforme; scFv, single-chain variable fragments; BiTE, bispecific T cell engager; CAR, chimeric antigen receptor; MAP, microtubule associated protein; TRAIL, TNF-related apoptosis-inducing ligand; HNSCC, head and neck squamous-cell carcinoma; CAR, chimeric antigen receptor; EGFR, epidermal growth factor receptor; EpCAP, epithelial cell adhesion molecule*.

While many treatment strategies centered on CSPG4 appear to have had success both *in vitro* and *in vivo* in rodent models, the next steps require in-depth studies with humanized or human antibodies, in disease-relevant and in clinically congruent models of cancer, including animal models engrafted with components of human immunity. These will permit mechanistic and efficacy evaluations in systems better able to recapitulate the patient setting.

An exciting prospect for targeting CSPG4 is the observed synergy between anti-CSPG4 monoclonal antibodies and BRAF inhibitors. In melanoma, a proportion of patients’ tumors have constitutively activated BRAF. Small molecule inhibitors recognizing mutant forms of BRAF have proved very effective. However, clinical responses are often short-lived due to the emergence of resistance. Combinatorial studies of monoclonal anti-CSPG4 antibodies with BRAF inhibitors have demonstrated enhanced effects and delayed the occurrence of resistance. Further understanding of the mechanisms that underpin the efficacy of these and other combinatory strategies may offer important clues that stand to improve current treatments.

Additionally, targeting CSPG4 may lead to targeted therapy for triple-negative breast cancer patients who do not benefit from therapies apart from standard chemotherapy. Therefore, further research and translation into clinical trials could be especially beneficial for the TNBC patient group.

As with all therapeutic approaches, the benefits of treatment must be balanced with the likelihood and severity of adverse effects. CSPG4 is expressed at low levels in some normal tissues; therefore, it is important to evaluate and mitigate any on-target, off-tumor toxic effects of CSPG4-specific targeted therapy. Encouragingly, a phase I clinical trial investigating anti-CSPG4 radioimmunotherapy with a mAb (9.2.27) conjugated to an α-particle-emitting radioisotope which was administered systemically in patients with melanoma reported no adverse events while some clinical benefits were observed (108). Furthermore, CSPG4-based immunotherapy strategies would benefit from the development of more effective methods of treatment delivery, such as hypobaric pressure skin delivery, which would limit potential off-target effects and reduce the cost of the therapy (120).

The emergence of novel antibody-based approaches offers fresh optimism that aggressive cancers, such as TNBC, glioma and head and neck carcinomas, which do not benefit from currently available therapies, but for which CSPG4 expression and its tumor-promoting functions have been reported, may become responsive to treatments based on this target. Therefore, renewed focus on CSPG4 may in future translate into significant benefits for patients with cancer.

## Author Contributions

KI, SK, and AT conceived and designed the study. KI, AC, SM, GC, SC, MG, MN searched and studied the literature. KI, MG, SK and AT wrote the manuscript. AC, SM, GC, MN, JS, ST, KE discussed and interpreted the literature findings and helped to edit the manuscript. SK supervised the study.

## Conflict of Interest Statement

SK and JS are founders and shareholders of IGEM Therapeutics Ltd. All other authors declare no conflicts of interest.
